# Acquired Gefitinib-Resistant Mutation of EGFR in a Chemonaive Lung Adenocarcinoma Harboring Gefitinib-Sensitive Mutation L858R

**DOI:** 10.1371/journal.pmed.0020269

**Published:** 2005-09-27

**Authors:** Chien-Hung Gow, Jin-Yuan Shih, Yih-Leong Chang, Chong-Jen Yu

**Affiliations:** **1**National Taiwan University HospitalTaipeiTaiwan

The research article by Pao et al. [1] provides important new information addressing three patients with acquired resistance to gefitinib or erlotinib in progressing tumors containing a secondary mutation, leading to the substitution of methionine for threonine at position 790 (T790M) in exon 20. However, all of the patients received systemic chemotherapy prior to gefitinib or erlotinib therapy, and the original lung tissue was obtained long before epidermal growth factor receptor (EGFR) inhibitors were used. We describe a chemonaive patient with gefitinib-sensitive lung adenocarcinoma harboring L858R. The tumor progressed and developed an additional T790M mutation after nine months of gefitinib treatment.

**Figure 1:**
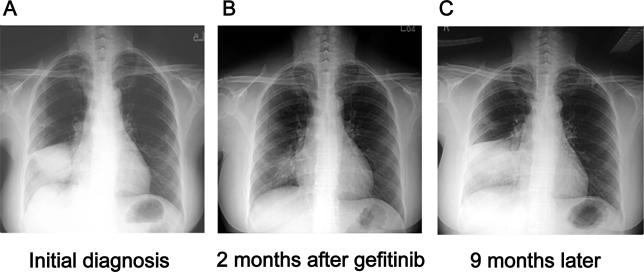
Chest Radiography Chest radiography shows a large mass in the RLL before gefitinib treatment (A), and marked decrease in tumor size two months after gefitinib was initiated (B). This tumor progressed nine months after gefitinib treatment (C).

A 56-year-old female who had never smoked presented with nonproductive cough for one month. Her chest radiography revealed a mass in the right lower lung (RLL) (Figure 1A). Chest tomography (CT) confirmed a mass with pleural invasion and multiple small nodules in the bilateral lungs. Ultrasound-guided percutaneous transthoracic lung biopsy revealed adenocarcinoma. Gefitinib (250 mg/day) was initiated. The RLL tumor decreased in size significantly two months after treatment (Figure 1B). Both serum CEA and CA-199 decreased, from 4,178 ng/ml to 9.1 ng/ml and from 464 U/ml to 22 U/ml, respectively. However, the patient could not tolerate the severe side effects, including diarrhea, erythematous papules over the nasolabial areas and buttocks, and paronychia with granulation on fingers. Gefitinib was withdrawn for two weeks. Then, she received gefitinib at 250 mg on alternate days. These side effects became tolerable, and gefitinib at 250 mg/day was resumed. Nine months after initiating gefitinib, chest radiography revealed progression of tumor (Figure 1C). At this time, chest CT revealed tumor progression with an endobronchial tumor in the right middle bronchus. Gefitinib was discontinued. After obtaining written, informed consent from the patient, a CT-guided lung biopsy specimen was obtained. Pathological analysis confirmed the presence of adenocarcinoma. This patient received subsequent chemotherapy for advanced lung cancer.

Genomic DNA was extracted from the tumor specimen of an original lung biopsy and a progressive tumor biopsy specimen. The tyrosine kinase domain (exons 18–21) was amplified and sequenced. Mutations were also checked against the corresponding DNA from blood lymphocytes at the diagnosis of lung cancer. The original diagnostic biopsy specimen contained a thymidine to guanine mutation at nucleotide 2573 of exon 21, resulting in L858R. In the second biopsy, an additional single-base change from cytosine to thymidine was identified at nucleotide 2369 in exon 20, resulting in T790M.

This report strengthens the evidence of T790M as an acquired gefitinib-resistant mutation. Gefitinib responsiveness results in large part from the drug's effective inhibition of essential antiapoptotic signals transduced by the mutant receptor, and L858R is the most commonly detected mutation [2–5]. The T790M mutation is rarely found in tumors from patients not treated with either gefitinib or erlotinib, and could be discovered only in progressing tumors, in addition to a primary drug-sensitive mutation in EGFR. A non-small-cell lung cancer cell line bearing both T790M and L858R mutations was approximately 100-fold less sensitive to gefitinib or erlotinib, and did not show inhibition of tyrosine phosphorylation in vitro [1].

Pao et al. and Kobayashi et al. identified four cases with lung adenocarcinoma harboring pre-existing mutations of EGFR as delL747–E749 plus A750P, delE746–A750, or delL747–S752, prior to the use of gefitinib or erlotinib [1,6]. All of them had exposure to previous systemic chemotherapies and took a small-molecule tyrosine kinase inhibitor as the second- or third-line therapy, then all acquired a second mutation T790M in the following months after disease progression. In the case of our patient, the patient received no prior systemic chemotherapy, and we identified an initial gefitinib-sensitizing L858R EGFR mutation, followed by a T790M mutation concomitantly with L858R in the biopsy taken from the growing tumor nine months after gefitinib use. Though it is unlikely that prior chemotherapy led to the development of T790M mutation, given the complexity of EGFR mutation, further studies are still required to elucidate the role of T790M mutation in the context of EGFR mutations.

*This correspondence was peer reviewed.*
